# BES-Driven Machine Learning Prediction of Future Energy Loads in Broiler Housing Under SSP Climate Scenarios in South Korea

**DOI:** 10.3390/ani16132097

**Published:** 2026-07-06

**Authors:** Jaeeun Kim, Kyeong-Seok Kwon, Soon-kun Choi, Jong-Bok Kim, Dong-Hwa Jang, Byeonghyeon Kim, Seungsoo Kim

**Affiliations:** 1 Smart Animal Environment Division, National Institute of Animal Science, Rural Development Administration, Wanju 55365, Republic of Korea; wodms@korea.kr (J.K.);; 2 Research Policy Planning Division, Research Policy Bureau, Rural Development Administration, Jeonju 54875, Republic of Korea; 3 Planning and Coordination Bureau, Rural Development Administration, Jeonju 54875, Republic of Korea

**Keywords:** standardized broiler house design, climate change scenario, heating and cooling loads, machine learning, building energy simulation

## Abstract

The increasing frequency of heatwaves and high-temperature events due to climate change intensifies heat stress and mortality risk in broiler production and necessitates reassessment of facility design and energy management. This study developed a machine learning surrogate model trained on BES-simulated heating and cooling loads to estimate future energy load changes in broiler houses under SSP climate scenarios. Training data were constructed using simulated energy load results under different insulation conditions combined with historical meteorological data (2011–2020). Four machine learning models, namely Linear Regression, Random Forest, Gradient Boosting, and XGBoost, were applied to compare their predictive performance for heating and cooling loads. Model performance was evaluated using five-fold cross-validation (K-fold cross-validation, K = 5). XGBoost and Random Forest showed the best performance for heating and cooling load prediction, respectively (R^2^ > 0.99, MAPE < 10%). These models were applied to SSP1-2.6 and SSP5-8.5 climate scenarios. Results showed decreasing heating loads and increasing cooling loads across all scenarios, with cooling demand projected to increase by more than 150% relative to baseline levels under SSP5-8.5 by the late 21st century. This study demonstrates that simulation-trained machine learning can efficiently estimate long-term energy-demand changes, supporting climate-responsive facility design and energy management in livestock housing.

## Simple Summary

Climate change is increasing heatwaves and extreme temperatures, raising heat stress and mortality risks in broiler production while changing heating and cooling energy demands. Predicting these changes is essential but complex. This study developed a machine learning-based model to efficiently estimate energy loads in broiler houses using historical weather data and simulation results. The selected models showed high prediction accuracy and revealed clear trends under future climate scenarios. Heating demand is expected to decrease, while cooling demand will increase significantly, especially under severe climate conditions. These findings can support better facility design, energy management, and climate-adaptive strategies in poultry production.

## 1. Introduction

The poultry industry has become one of the most important sources of animal protein worldwide and has experienced continuous growth with the expansion of intensive production systems. However, environmental changes associated with climate change, including rising average temperatures and the increasing occurrence of extreme temperature events, have been identified as major factors that increase environmental management burdens and operational uncertainty in poultry production systems [1]. In South Korea, chicken consumption has steadily increased with changing dietary patterns characterized by a growing preference for chicken cuts. In 2023, per capita chicken consumption reached 16.2 kg, representing a 9.5% increase compared with the previous year (14.8 kg) [2]. Nevertheless, despite the increasing demand for chicken meat, the domestic broiler industry faces growing challenges, including labor shortages due to population aging and rising production costs. In addition to these challenges, environmental pressures associated with climate change further undermine the operational stability of the industry.

The frequency of heatwaves has gradually increased due to climate change, and the resulting heat stress can lead not only to reduced average daily gain (ADG) but also to increased mortality in broilers [3,4,5,6]. This phenomenon is observed globally rather than being limited to a specific region [1,7,8]. In particular, productivity losses associated with thermal stress may be more pronounced in intensive poultry housing systems. In South Korea, the national average number of heatwave days reached 31 days in 2018, the longest duration since meteorological observations began, resulting in the mortality of approximately 8.33 million poultry birds [9,10]. In 2024, the national average number of heatwave days reached 30.1, and the annual mean maximum temperature recorded 19.7 °C, representing the second longest heatwave duration and the highest annual mean maximum temperature on record [9]. According to climate projections by the Korea Meteorological Administration, the annual mean temperature of the Korean Peninsula is projected to increase by up to 7 °C by the late 21st century (2081–2100) compared with the present baseline period (1995–2014), depending on the Shared Socioeconomic Pathways (SSP) scenarios [11,12]. These projections suggest that the increasing occurrence of extreme climate events may contribute to increased environmental management challenges in broiler housing systems. In addition, in broiler production, both cooling during hot periods and heating during colder periods are critical environmental management factors. Maintaining optimal brooding temperatures for young chicks requires substantial heating energy when outdoor temperatures are low. As average temperatures increase due to climate change, cooling demand is expected to increase while heating demand may decrease, highlighting the need for systematic thermal environment management in broiler houses under changing climatic conditions.

Effective thermal environment management in broiler houses requires appropriate capacity design and operational strategies for heating and cooling systems. To achieve this, it is necessary to first estimate the magnitude of energy loads required for broiler housing facilities. In general, building energy simulation (BES) techniques are widely used to predict building energy loads, as they can estimate energy demand by incorporating building design specifications and regional climate conditions [13,14].

Previous studies have applied BES and computational fluid dynamics (CFD) approaches to analyze the thermal environment in poultry facilities, reporting energy load variations and heat stress responses under different stocking densities, building envelope conditions, and ventilation characteristics [15,16,17,18]. In particular, Kwon et al. (2026) [19] developed a BES-based energy load prediction algorithm for broiler houses based on the Korean Livestock House Design Standards. The model was developed to predict energy loads while reflecting regional climate conditions, building specifications, housing capacity, and insulation characteristics, providing a framework for scientifically quantifying energy demand in broiler housing systems in South Korea.

As noted above, rising average temperatures and the increasing occurrence of extreme climate events are expected to intensify environmental challenges in broiler production systems. In this context, analyzing the variability of energy loads in livestock housing under future climate conditions is crucial for developing strategies to mitigate heat stress while maintaining productivity and operational stability. However, previous studies have primarily focused on specific climatic conditions or short-term future projections [14,15], and studies comparing energy demand changes across multiple regions using repeated applications of future climate scenarios remain limited. Although BES-based approaches provide precise estimates of energy demand under specific design conditions, repeatedly calculating energy loads across multiple regions and long-term future periods using future climate scenarios may require substantial computational time and cost. To address this limitation, machine learning–based data-driven models have recently been increasingly adopted in building energy demand prediction to reduce the computational burden associated with physics-based simulations [20,21]. These models can learn the relationships between complex input variables and energy loads, enabling rapid and scalable predictions for large-scale climate scenarios and diverse design conditions.

In this study, machine learning surrogate models were developed to predict heating and cooling energy loads in broiler houses using simulation outputs from the BES model developed by Kwon et al. (2026) [19]. Given the limited availability of standardized long-term measured energy-use data from commercial broiler farms, the validated BES outputs were used as a consistent training dataset for surrogate modeling. The developed model was then applied to SSP-based future climate scenarios to analyze long-term changes in energy loads across major regions in South Korea. Through this integrated BES-machine learning framework, this study enables large-scale analysis of regional variations in energy demand under future climate conditions, thereby providing a practical basis for climate-responsive insulation design, cooling management strategies, and broiler housing design standards.

## 2. Materials and Methods

### 2.1. Target Facility

**Figure 1:**
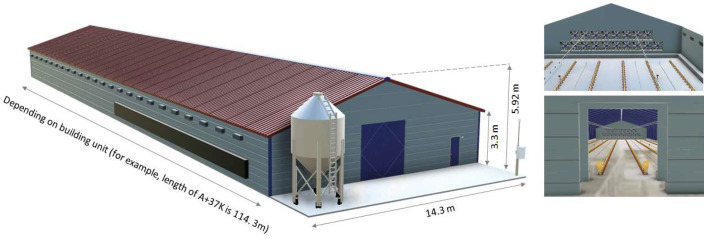
Schematic view of *A + 37K* broiler house of Livestock House Design Standards in Korea (Kwon et al., 2026 [19]).

In this study, future energy loads of broiler housing facilities were predicted based on a mechanically ventilated broiler house design specified in the Livestock House Design Standard (2016) issued by the Ministry of Agriculture, Food and Rural Affairs (MAFRA), which represents the most widely applied design guideline in practical farming conditions. The Livestock House Design Standard provides architectural guidelines for livestock housing as well as operational recommendations for ICT equipment, and it has been widely used to improve the efficiency of livestock facility design and management [19,22]. Broiler houses based on this standard typically adopt a closed-type structure with mechanical ventilation. During the summer season, tunnel ventilation is applied, whereas cross ventilation using sidewall exhaust fans and multiple air inlet baffles is operated during transitional seasons and winter. The standard design guidelines provide a total of 28 standard housing layouts depending on the size of broiler facilities. In this study, the A + 37K model was selected as the target facility, which can accommodate up to 39,485 broilers when applying the stocking density of 39 kg m^−2^ specified in the Livestock Act (Figure 1). The A + 37K house has a width of 14.3 m, a length of 114.3 m, a sidewall height of 3.3 m, and a roof height of 5.92 m. This facility configuration was used as the reference model for subsequent energy load simulations.

### 2.2. Study Areas

Following the approach of Kwon et al. (2026) [19], this study selected the study areas as administrative districts with the highest number of broilers in 2020, excluding special and metropolitan cities [23]. The selected study areas are listed in Table 1, including Cheorwon-gun (Gangwon-do, with 1,184,678 heads), Yangpyeong-gun (Gyeonggi-do, with 2,431,680 heads), Chungju-si (Chungcheongbuk-do, with 978,870 heads), Nonsan-si (Chungcheongnam-do, with 2,899,189 heads), Namwon-si (Jeollabuk-do, with 5,172,657 heads), Youngam-gun (Jeollanam-do, with 2,885,094 heads), Youngju-si (Gyeongsangbuk-do, with 7,454,525 heads), Sancheong-gun (Gyeongsangnam-do, with 1,344,562 heads), and Jeju-si (Jeju-do, with 923,774 heads).

### 2.3. Building Energy Simulation (BES) for Calculating Heating and Cooling Loads of Broiler Houses

**Figure 2:**
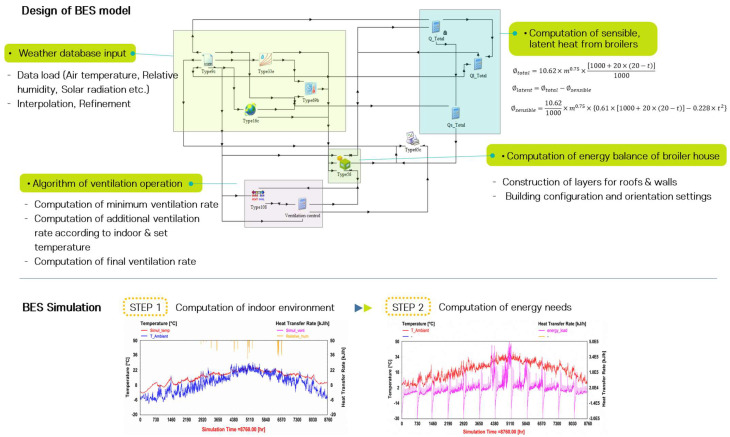
Methodology for computing energy loads in a broiler house under various simulation conditions. The upper diagram, adapted from Kwon et al. (2026) [19], shows the overall Building energy simulation (BES) modeling framework. The lower graphs, newly generated in this study, illustrate the two-step calculation procedure: dynamic simulation of indoor thermal conditions and subsequent calculation of heating and cooling energy loads required to maintain the target indoor environment.

In this study, the heating and cooling energy loads under future climate change scenarios were estimated using the energy load calculation results obtained from the BES model for broiler houses developed in a previous study [19]. This section briefly summarizes the key features of the BES model. Kwon et al. (2026) [19] developed a dynamic Building Energy Simulation (BES) model capable of estimating energy loads in broiler houses by comprehensively reflecting outdoor environmental conditions as well as facility and production characteristics. The model was developed based on the Livestock House Design Standard in Korea and was implemented using TRNSYS (Version 18, Solar Energy Laboratory, University of Wisconsin—Madison, WI, USA) (Figure 2). The BES procedure consisted of two sequential steps. In the first step, indoor thermal conditions were dynamically simulated by accounting for broiler production cycles, age-dependent body weight changes [24], ventilation requirements, and outdoor environmental conditions. In the second step, the heating and cooling energy loads required to maintain appropriate indoor thermal conditions were calculated on an hourly basis from the simulated thermal responses. The lower graphs in Figure 2 illustrate this two-step calculation process. In the previous study, meteorological data from 2011 to 2020 for each study area were used as input data. Energy loads were calculated under a total of 17,280 simulation conditions considering broiler house size, building orientation (0°, 45°, 90°, and 135°, considering building symmetry), roof insulation (three thickness conditions), and wall insulation (four thickness conditions). The performance of the developed model was reported with an NMBE of 0.78%, a CVRMSE of 9.75%, and a MAPE of 3.82%. By utilizing a validated BES model, a reliable simulation-based data generation framework was established, which contributes to improving the robustness and applicability of the subsequent machine learning-based energy load prediction. In the present study, simulation results corresponding to the A + 37K house type with an east–west building orientation were extracted from the previous study and used as training data for the machine learning–based energy load prediction model described in the subsequent sections.

### 2.4. Climate Change Scenarios and Global Climate Models

The Shared Socioeconomic Pathways (SSP) scenarios are the framework for future climate change projections adopted in the Sixth Assessment Report of the Intergovernmental Panel on Climate Change (IPCC) [25]. The SSP framework classifies future climate pathways into four scenarios according to the level of greenhouse gas mitigation and the implementation of climate adaptation strategies under different socioeconomic development trajectories [25]. The scenarios ranging from SSP1-2.6 to SSP5-8.5 represent contrasting development pathways, from a sustainable low-carbon society to an energy-intensive society with high dependence on fossil fuels.

The SSP climate scenarios are commonly applied to Global Climate Models (GCMs) developed by various countries and research institutions under the Climate Model Intercomparison Project Phase 6 (CMIP6) framework. Because GCMs differ in model structure, spatial resolution, and representation of physical processes, their ability to reproduce regional climate conditions also varies [26]. These differences can result in varying future climate projections even under the same emission scenario. The Korea Meteorological Administration (KMA) climate information portal provides ensemble mean values from 18 GCMs participating in CMIP6 [27]. However, such ensemble averages may not fully capture the variability of climatic characteristics that are critical for predicting energy loads, particularly extreme temperature conditions. In the case of heating and cooling load estimation in livestock housing, the maximum or minimum outdoor temperature has a substantial influence on energy demand. When models that do not adequately reflect regional climatic characteristics are applied, the resulting projections may show relatively low accuracy and limited representation of extreme values. The KACE-1-0-G model is a Korean Earth system model developed by the National Institute of Meteorological Sciences (NIMS) to reflect the climatic characteristics of the Korean Peninsula, whereas the UKESM1-0-LL model is a widely used GCM known for its ability to simulate temperature and climate variables by comprehensively representing interactions within the Earth system [28,29,30]. Therefore, in this study, two GCMs with different structural characteristics and relatively good performance in representing regional climate conditions were selected to estimate energy loads while considering structural uncertainties among climate models.

**Figure 3:**
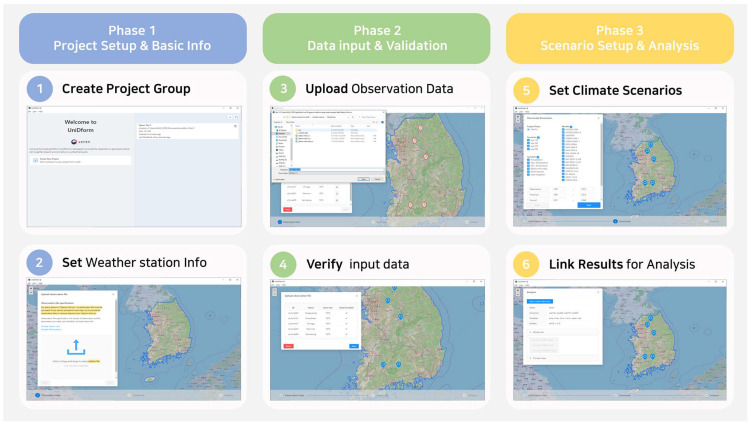
Workflow of the climate scenario utilization platform (UNIDFORM) developed by the National Institute of Agricultural Sciences.

Future climate data were generated using UNIDFORM (Climate Scenario Utilization Platform, National Institute of Agricultural Sciences, Rural Development Administration, Joenju, Republic of Korea), a climate change scenario application platform [31]. Following the procedure shown in Figure 3, daily future climate data for each study area were generated for the period from 2011 to 2100 under the SSP1-2.6 and SSP5-8.5 scenarios. This study aimed to consider uncertainties in climate change associated with different greenhouse gas emission pathways and to compare energy demand changes under contrasting climate change scenarios. Although the generated climate dataset included daily maximum and minimum air temperature, precipitation, wind speed, relative humidity, and solar radiation, these variables were not all used directly as final inputs to the machine learning surrogate model. The final meteorological inputs were selected in Section 2.5 after considering data consistency, temporal resolution, and reliability for long-term SSP-based application.

### 2.5. Dataset Construction for Machine Learning Application

**Figure 4:**
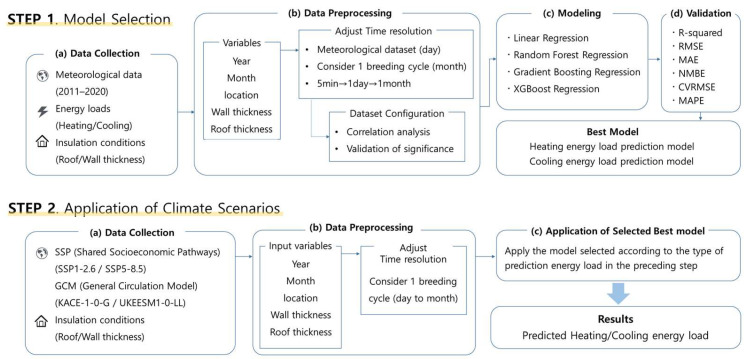
Flowchart of the heating and cooling energy-load prediction model. Arrows indicate the sequential workflow from data collection and preprocessing to model development, validation, and application to future climate scenarios. SSP, Shared Socioeconomic Pathways; GCM, Global Climate Model; RMSE, root mean square error; MAE, mean absolute error; NMBE, normalized mean bias error; CVRMSE, coefficient of variation of the root mean square error; MAPE, mean absolute percentage error.

The overall workflow for dataset preparation and model development for machine learning–based energy load prediction is illustrated in Figure 4. The data preparation process described in this section is based on the procedures presented in Section 2.3 and Section 2.4, while the machine learning model configuration and validation are described in Section 2.6.

In the data collection stage, solar radiation was limited to the daily maximum and minimum values provided by UNIDFORM. Therefore, to ensure the reliability of the input variables, this study limited the meteorological inputs to temperature-related variables. The insulation conditions and corresponding energy load values were adopted from the previous study by Kwon et al. (2026) [19].

Although non-temperature meteorological variables such as relative humidity, wind speed, precipitation, and solar radiation may influence building energy loads, this study prioritized temperature-derived variables to maintain consistency between the historical BES-based training dataset and the long-term SSP climate scenario dataset. In addition, ventilation requirements, broiler production cycles, age-dependent body weight changes, and associated internal heat generation were already reflected in the BES-simulated energy loads used as target outputs. Therefore, the ML surrogate model focused on consistently available temperature-related variables that directly represent outdoor thermal conditions affecting heating and cooling demand.

In Step 1, preprocessing and analysis were conducted to construct a dataset suitable for machine learning by combining observed meteorological data with the BES-based calculated heating and cooling energy loads. First, the temporal resolution of the datasets was standardized. The historical meteorological observations and BES results used for calculating energy loads were recorded at 5 min intervals, whereas the future meteorological data generated from the SSP scenarios were provided at daily intervals. To ensure consistency in the input data structure, the 5 min interval temperature observations were converted into daily maximum and minimum temperatures.

In Step 2, considering that the average broiler production cycle in South Korea is approximately 30 days, the dataset was reconstructed on a monthly scale to represent the overall variations in thermal conditions during the production period. From the daily temperature data, the monthly mean and standard deviation of daily maximum and minimum temperatures were derived (tmax_avg, tmax_std, tmin_avg, tmin_std). Based on these values, the final input variables were constructed, including monthly mean temperature (avg_temp), the monthly mean diurnal temperature range (range_temp = tmax_avg − tmin_avg), and the coefficients of variation for daily maximum and minimum temperatures (tmax_CV = tmax_std/tmax_avg, tmin_CV = tmin_std/tmin_avg). The monthly mean temperature variable was used to represent the baseline thermal conditions during the corresponding period, while the monthly mean diurnal temperature range was used to capture daily temperature variability. The coefficients of variation represent temperature variability within a given period and were included to indirectly reflect the potential occurrence of extreme temperature events, such as heatwaves, and the instability of the thermal environment. In accordance with the input variable structure, the heating and cooling energy loads were recalculated as monthly totals based on the original 5 min interval simulation outputs.

After standardizing the temporal resolution of the datasets, the meteorological conditions (temperature variables) and insulation conditions were used as input variables, and the calculated energy loads served as output variables. Because high correlations among input variables may cause multicollinearity and distort prediction results, the dataset was constructed to minimize correlations among temperature variables during the temporal aggregation process. Prior to model training, correlation analysis, significance testing (*p*-value), and analysis of variance (ANOVA) were conducted to evaluate the suitability of the dataset structure. The results indicated that major temperature variables and insulation conditions significantly influenced heating and cooling energy loads, and that seasonal factors and wall insulation conditions played important roles in energy load variability. Temporal variables including Year and Month were introduced to simultaneously reflect long-term climate trends and seasonal variability. The Month variable was used directly as a numerical value without separate seasonal categorization to preserve both seasonal continuity and monthly characteristics.

Instead of using region names as categorical variables, the latitude and longitude of the meteorological observation stations were used as input variables to facilitate potential future expansion of the prediction model. The insulation condition data were applied uniformly across the dataset without additional preprocessing. The final classification of input and output variables in the constructed dataset is summarized in Table 2.

### 2.6. Machine Learning Model Design

In this study, four machine learning regression models commonly used for energy load prediction were applied to compare their predictive performance for heating and cooling loads [32,33,34]. The models considered were Linear Regression, Random forest, Gradient Boosting, and Extreme Gradient Boosting (XGBoost). The models were trained using observed meteorological data (2011–2020) and insulation thickness as input variables and corresponding energy load outputs simulated by the BES model. Based on performance evaluation, the best-performing model was selected and used to predict energy loads under future climate conditions.

The objective of this study was not to derive the optimal performance of a specific algorithm but rather to compare the predictive performance of different machine learning algorithms and evaluate their potential as data-driven prediction models that can complement BES-based analysis. To ensure fair comparison among algorithms, the models were trained using the default hyperparameters provided by each library. For the Random forest and Gradient Boosting regression models, the parameter was set to n_estimators = 100, while the XGBoost regression model used n_estimators = 100 and learning rate = 0.1. The Linear Regression, Random forest, and Gradient Boosting models were implemented using scikit-learn v1.3.0, whereas the XGBoost model was trained using the XGBoost v2.1.4 library. 

Model performance was evaluated using five-fold cross-validation (K-fold cross-validation, K = 5). The entire dataset was divided into five subsets, and in each iteration, four subsets were used for training (train dataset) while the remaining subset was used for validation (validation dataset). This process was repeated five times so that all subsets were used once for validation. The final performance metrics were obtained by averaging the results from all iterations.

Model performance was assessed using R^2^, RMSE, and MAE, as well as NMBE, CVRMSE, and MAPE, which are recommended by ASHRAE Guideline 14 for evaluating building energy simulation models. These metrics were used to compare the prediction accuracy and performance of the models. The practical applicability of the models was also examined based on the criteria suggested in ASHRAE Guideline 14 (NMBE < 10%, CVRMSE < 30%, MAPE < 20%). Each performance metric can be calculated as shown in Equations (1)–(6).

(1) $$R^{2}=1-\frac{{SS}_{res}}{{SS}_{tot}}$$

(2) $$MAE=\frac{1}{n}\sum_{i=1}^{n}\left|y_{i}-{\hat{y}}_{i}\right|$$

(3) $$RMSE=\sqrt{\frac{1}{n}\sum_{i=1}^{n}{\left(y_{i}-{\hat{y}}_{i}\right)}^{2}}$$

(4) $$CVRMSE=\frac{RMSE}{\bar{y}}\times 100$$

(5) $$MAPE=\frac{100}{n}\sum_{i=1}^{n}\left|\frac{y_{i}-{\hat{y}}_{i}}{y_{i}}\right|$$

(6) $$NMBE=\frac{\sum_{i=1}^{n}(y_{i}-{\hat{y}}_{i})}{\sum_{i=1}^{n}y_{i}}\times 100$$

where ${SS}_{res}=\sum {\left(y_{i}-{\hat{y}}_{i}\right)}^{2},{SS}_{tot}=\sum {\left(y_{i}-\bar{y}\right)}^{2}$; $y_{i}$ is the observe value, ${\hat{y}}_{i}$ is the predicted value, ${\bar{y}}_{i}$ is the mean of the observed values, and n is the number of observations. The specifications of the computer used for model training and analysis are presented in Table 3.

## 3. Results

### 3.1. Performance of Machine Learning Models for Heating and Cooling Load Prediction

The performance evaluation of the heating load prediction models showed consistent ranking across all evaluation metrics, with XGBoost, Random Forest, Gradient Boosting, and Linear Regression in descending order of performance. In terms of the coefficient of determination (R^2^), Linear Regression achieved a value of 0.915, whereas XGBoost (0.999), Random Forest (0.998), and Gradient Boosting (0.997) showed substantially higher explanatory power, indicating the superior predictive capability of tree-based ensemble models compared with the linear regression model. A similar pattern was observed in the prediction error metrics. The RMSE was lowest for XGBoost (4.34), followed by Random Forest (6.42), Gradient Boosting (6.98), and Linear Regression (38.98), confirming the superior performance of ensemble models. The MAE values also followed the same trend, with XGBoost (3.20), Random Forest (4.37), Gradient Boosting (5.29), and Linear Regression (29.01), indicating that ensemble models provided more stable predictions of actual heating load values. Similarly, the MAPE values were 1.59%, 1.87%, 2.62%, and 14.10% for XGBoost, Random Forest, Gradient Boosting, and Linear Regression, respectively, further demonstrating the stable prediction performance of the models. These results represent the mean values obtained from five-fold cross-validation, and the standard deviations of the evaluation metrics were very small for all tree-based ensemble models. This indicates that model performance remained consistent across different cross-validation folds and that the models achieved stable predictive performance for heating load estimation regardless of data partitioning.

In addition, the model was evaluated using NMBE and CVRMSE based on the criteria suggested in ASHRAE Guideline 14. The results showed that all nonlinear models satisfied these criteria, with NMBE within ±10% and CVRMSE below 30%, indicating that the models achieved prediction accuracy suitable for practical implementation.

For the cooling load prediction models, the performance ranking was Random Forest, XGBoost, Gradient Boosting, and Linear Regression. In terms of R^2^, Random Forest achieved 1.000, followed by XGBoost (0.999) and Gradient Boosting (0.989), all showing strong explanatory power. In contrast, Linear Regression showed a substantially lower R^2^ value of 0.581, indicating a significant decline in predictive capability for cooling load estimation. The RMSE values were relatively large for Linear Regression (145.45) and Gradient Boosting (24.10), whereas XGBoost (7.63) and Random Forest (4.18) showed much smaller prediction errors. A similar pattern was observed for the MAE metric. Random Forest (1.41) and XGBoost (4.44) produced substantially lower absolute errors than Linear Regression (101.67). The results from five-fold cross-validation also showed relatively small standard deviations for Random Forest and XGBoost across most evaluation metrics, indicating consistent performance across validation folds. When evaluated against the practical applicability criteria, all nonlinear models satisfied the CVRMSE threshold, whereas Linear Regression did not. However, in terms of MAPE, only the Random Forest model satisfied the recommended threshold. The Random Forest showed the most stable performance for cooling load prediction in terms of both mean error and relative error.

Overall, XGBoost regression showed the best performance for heating load prediction, while Random Forest performed best for cooling load prediction. Accordingly, these two models were selected for predicting future energy loads using climate change scenarios. The performance evaluation results for the heating and cooling load models are summarized in Table 4 and Table 5, respectively, while the performance metrics for each cross-validation fold are provided in Appendix A (Table A1 and Table A2).

### 3.2. Future Energy Load Prediction Under GCM-Based Climate Scenarios

**Figure 5:**
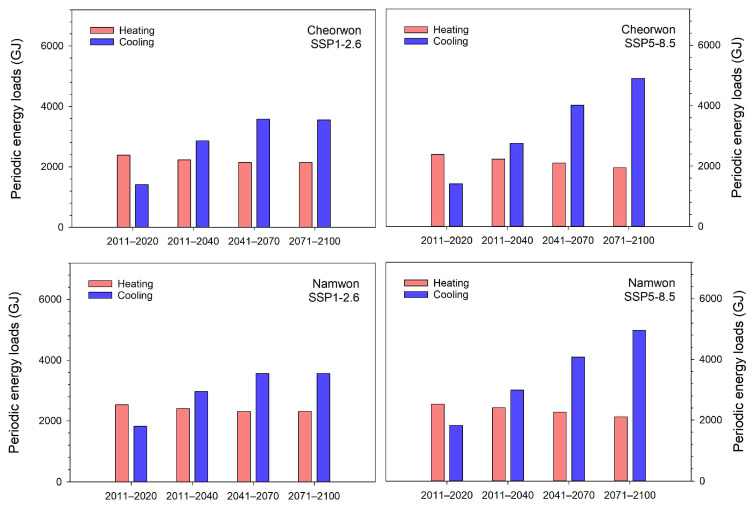
Periodic heating and cooling energy loads in Cheorwon and Namwon under the SSP1-2.6 and SSP5-8.5 climate scenarios derived from the KACE-1-0-G GCM model. Bars represent heating and cooling loads (GJ) for four analysis periods: 2011–2020, 2011–2040, 2041–2070, 2071–2100.

**Figure 6:**
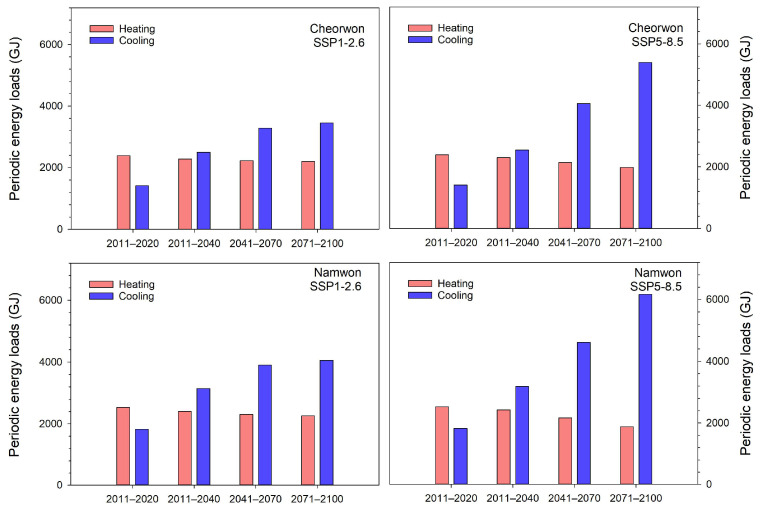
Periodic heating and cooling energy loads in Cheorwon and Namwon under the SSP1-2.6 and SSP5-8.5 climate scenarios derived from the UKESM1-0-LL GCM model. Bars represent heating and cooling loads (GJ) for four analysis periods: 2011–2020, 2011–2040, 2041–2070, 2071–2100.

The heating and cooling energy loads calculated for the baseline period (2011–2020) using the BES model were used as reference values to analyze future changes in energy demand. Based on the model performance results, the XGBoost model was applied for heating load prediction and the Random Forest model for cooling load prediction. Future energy loads were estimated under SSP climate scenarios using the KACE-1-0-G and the UKESM1-0-LL GCM. Among the nine study regions, results are presented for Cheorwon and Namwon, which represent different climatic zones. In South Korea, building energy standards classify regions into four climate zones (Central 1, Central 2, Southern, and Jeju). Cheorwon is classified as Central 1, while Namwon belongs to Central 2 (Table 6). These regional classifications determine the minimum insulation performance required for building envelopes and floors [19,35]. In this study, energy loads were calculated based on the minimum insulation design criteria specified by the Ministry of Land, Infrastructure and Transport. The applied insulation thicknesses were 190 mm (wall), 220 mm (roof) for Central 1 regions, 135 mm and 220 mm for Central 2 regions, 100 mm and 180 mm for Southern regions, and 75 mm and 130 mm for Jeju. Under these conditions, the predicted heating and cooling energy loads for the periods 2011–2040, 2041–2070, and 2071–2100 are shown in Figure 5 and Figure 6.

For the baseline period (2011–2020), Cheorwon showed a heating load of 2382.5 GJ and a cooling load of 1412.2 GJ, indicating that heating demand was higher than cooling demand. Under the SSP1-2.6 scenario, the heating load gradually decreased over time and was projected to reach 2139.1 GJ in 2071–2100, representing a 10.2% reduction from the baseline period. In contrast, the cooling load continuously increased and exceeded 3552.9 GJ in 2071–2100, corresponding to an increase of more than 150% relative to the baseline period. Under the SSP5-8.5 scenario, the reduction in heating load was larger, reaching 1947.8 GJ in 2071–2100 (18.2% decrease). Meanwhile, the cooling load increased to 4903.6 GJ, corresponding to a 247.2% increase compared with the baseline period.

A similar pattern was observed for Namwon. During the baseline period (2011–2020), the heating load was 2528.1 GJ, and the cooling load was 1827.3 GJ, both higher than those observed in Cheorwon. In 2071~2100, the heating load was projected to decrease to 2315.7 GJ (8.4% reduction) under SSP1-2.6 and 2106.1 GJ (16.7% reduction) under SSP5-8.5. In contrast, the cooling load increased to 3563.9 GJ and 4954.6 GJ, representing increases of 95.0% and 171.1%, respectively. In the period 2071–2100, the rapid increase in cooling load in Cheorwon reduced the regional difference in cooling demand between Cheorwon and Namwon to approximately 1%.

When the UKESM1-0-LL GCM was applied, trends similar to those obtained from the KACE-1-0-G GCM were observed Under the SSP1-2.6 scenario, the projected heating load in Cherowon decreased by 4.3% (2279.7 GJ) in 2011–2040, 6.7% (2221.8 GJ) in 2041–2070, and 7.8% (2196.8 GJ) in 2071–2100 relative to the baseline period. During the same periods, the cooling load increased by 76.7% (2494.8 GJ), 132.4% (3281.5 GJ), and 144.4% (3451.6 GJ), respectively. Although the rate of increase was slightly lower than that obtained using KACE GCM, the cooling load increase in Cheorwon remained relatively large compared with other regions, showing a similar overall trend. Under the SSP5-8.5 scenario, the cooling load in Cheorwon increased by 79.8% (2538.8 GJ), 187.2% (4056.1 GJ), and 281.7% (5390.9 GJ) during the three future periods, showing a steep increase relative to the baseline period. In general, both GCMs showed a larger increase in cooling load compared with the reduction in heating load, and this difference became more pronounced under the SSP5-8.5 scenario.

**Figure 7:**
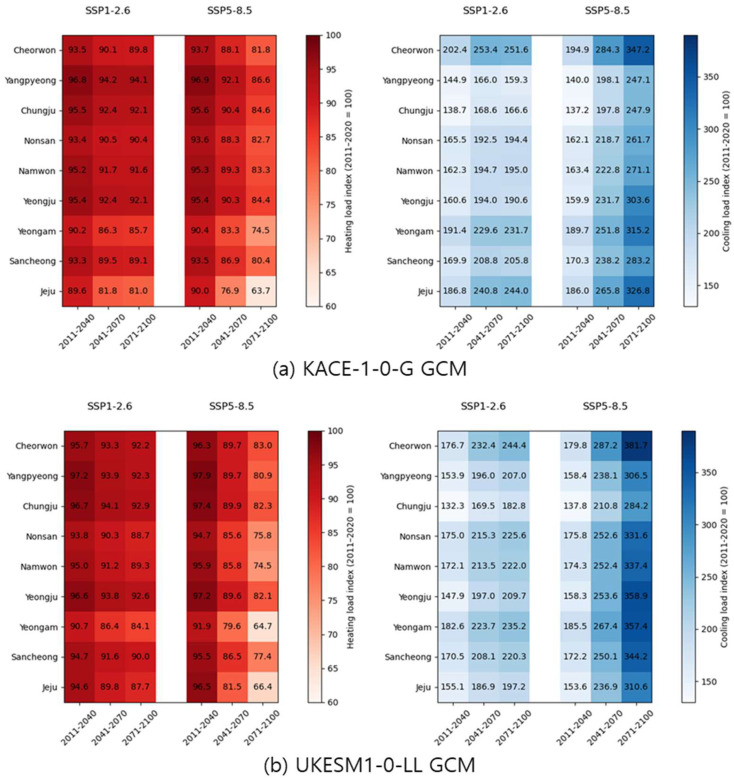
Relative changes in heating (**left**) and cooling (**right**) energy loads under SSP1-2.6 and SSP5-8.5 scenarios simulated using two GCMs: (**a**) KACE-1-0-G and (**b**) UKESM1-0-LL. Regional minimum insulation standards were applied (see Table 6 for details). Energy loads are presented as indices normalized to the 2011–2020 baseline (2011–2020 = 100).

In addition, the relative changes in heating and cooling loads compared with the baseline period (2011–2020) were normalized as indices to analyze the overall patterns of future energy load changes (Figure 7). The results showed clear differences between the two SSP scenarios. Under the SSP1-2.6 scenario, heating loads gradually decreased while cooling loads increased across most regions, with the magnitude of change becoming relatively moderate after around 2070. In contrast, under the SSP5-8.5 scenario, the decrease in heating loads and the increase in cooling loads continued to intensify throughout the entire projection period.

Regional comparisons showed that under the SSP5-8.5 scenario, the variability in cooling load increases and heating load reductions among regions became larger toward the mid- and late-century periods. In particular, the cooling load in Cheorwon increased substantially in the long-term projection period, whereas the heating load in Jeju showed a relatively clear decreasing trend. These results showed that the magnitude and direction of future energy demand changes vary depending on regional climate characteristics.

In the comparison between GCMs, the UKESM1-0-LL GCM showed differences in the absolute magnitude of heating and cooling load changes compared with the KACE-1-0-G, although the overall trends were similar. In particular, under the SSP5-8.5 scenario after 2040, the UKESM model projected larger reductions in heating loads and larger increases in cooling loads across most regions compared with the KACE. In addition, detailed predicted heating and cooling loads for all regions, GCMs, SSP scenarios, and future periods are provided in Supplementary Tables S1–S4.

## 4. Discussion

Before interpreting the model performance, the scope of the proposed framework should be clarified. The machine learning model developed in this study was designed as a surrogate model for BES-based energy-load estimation. Therefore, the reported prediction accuracy represents the agreement between the machine learning outputs and the BES-simulated heating and cooling loads, rather than validation against measured energy use in commercial broiler farms. This approach was adopted because standardized long-term measured energy load data from broiler houses are limited and can be strongly affected by differences in housing structure, equipment specifications, and farm management practices. The BES model used as the data source had previously satisfied commonly accepted performance criteria, including NMBE, CVRMSE, and MAPE, thereby providing a reliable simulation dataset for surrogate modeling. Although this validation cannot completely eliminate the possibility that limitations of the BES model are reflected in the surrogate model outputs, it reduces this concern by using a previously evaluated simulation model as the data source. Nevertheless, because the target outputs were generated from a simulation model, the surrogate model outputs should be interpreted as BES-based energy-load estimates rather than direct measurements of farm energy use. As standardized measured energy-use data from commercial broiler farms become available, future studies should further evaluate the transferability of the proposed surrogate framework under real farm conditions.

The use of temperature-related variables as the final meteorological inputs should also be considered when interpreting the scope of the present surrogate modeling framework. Although relative humidity, wind speed, precipitation, and solar radiation may directly or indirectly affect thermal boundary conditions and building energy loads, this study prioritized temperature-derived variables to maintain consistency and reliability between the historical BES-based training dataset and the long-term SSP climate scenario dataset. In addition, ventilation requirements, broiler production cycles, age-dependent body weight changes, and associated internal heat generation were reflected in the BES-simulated heating and cooling loads used as target outputs. Therefore, the present model mainly represents the response of BES-based energy-load estimates to temperature-related climate changes and insulation conditions. Although monthly mean temperature, diurnal temperature range, and coefficients of variation were used to indirectly reflect thermal variability within each month, monthly aggregation may not fully capture the intensity, duration, and frequency of short-term extreme temperature events such as heatwaves. Future studies should extend this framework by incorporating high-resolution and consistently defined climate variables and by evaluating their additional contribution to prediction accuracy when standardized measured energy-use data from commercial broiler farms become available.

Within this defined scope of the BES-based surrogate modeling framework, XGBoost showed the best performance for estimating heating loads, whereas Random Forest showed the best performance for estimating cooling loads. Both models satisfied the criteria suggested in ASHRAE Guideline 14, demonstrating statistical accuracy and supporting their applicability within the proposed framework. The selection of different optimal models for heating and cooling load estimation can be interpreted as reflecting differences between the characteristics of heating and cooling load variations and the structural properties of the machine learning algorithms.

The very high R^2^ values obtained in this study should be interpreted in relation to the structure of the dataset. The training and validation data were generated from a deterministic BES model under standardized combinations of region, month, insulation conditions, and meteorological variables. As a result, the input-output relationships were more structured and internally consistent than those typically found in measured farm energy-use data. Tree-based ensemble models, such as Random Forest and XGBoost, are well suited to learning nonlinear relationships and repeated patterns in tabular datasets, which likely contributed to the near-perfect R^2^ values observed in this study. Therefore, the high R^2^ values primarily indicate that the proposed models effectively reproduced the structured patterns of BES-simulated heating and cooling loads within the constructed dataset. Although five-fold cross-validation confirmed the reproducibility of BES-simulated energy load patterns within the constructed dataset, it does not fully verify model transferability to unseen regions, unseen climate-insulation combinations, or long-term future climate conditions. In addition, because tree-based models have limited extrapolation capability beyond the range of the training data, the role of temporal variables should be interpreted carefully. Future studies should further evaluate the robustness of the proposed framework using temporal split validation, leave-one-region-out validation, and tests on unseen climate or insulation combinations.

In this study, the heating load showed a relatively gradual decreasing trend and smaller variability compared with the cooling load. As a boosting-based algorithm that sequentially corrects residual errors, XGBoost is well suited to learning gradual change patterns in the data, which likely contributed to its superior performance in heating load prediction. In contrast, the cooling load exhibited greater variability and relatively rapid growth during certain periods. This pattern suggests that cooling energy demand is influenced not only by mean temperature but also by variability in temperature-related variables, such as maximum temperature and temperature ranges. Because Random Forest is effective at capturing nonlinear relationships and interactions among variables, it may have been better suited for predicting cooling loads characterized by complex and highly variable patterns. In addition, cooling loads often exhibit threshold-based behavior in which energy demand increases rapidly once temperatures exceed a certain level. Such nonlinear patterns may be effectively captured by tree-based algorithms such as Random Forest. Although XGBoost and Gradient Boosting showed high explanatory power in terms of R^2^ for cooling load prediction, their MAPE values were relatively large. This result is likely due to the presence of periods during winter when cooling loads are extremely low or close to zero, which can inflate relative error-based metrics such as MAPE. Overall, these results suggest that the performance of a particular machine learning model in energy load prediction may depend on the physical characteristics of the target phenomenon and the distributional structure of the data. This finding implies that, in the development of energy management models for livestock facilities, the selection of machine learning algorithms should be based not only on predictive accuracy but also on an understanding of the underlying mechanisms governing energy load generation.

The heating and cooling energy loads in Namwon and Cheorwon were compared in the previous section. In general, Cheorwon, located at a higher latitude than Namwon, experiences relatively lower ambient temperatures, which would normally lead to higher heating demand. However, in this study, both heating and cooling loads during the baseline period were higher in Namwon than in Cheorwon. This difference may be attributed to variations in building insulation conditions among regions, which influence the estimation of energy loads. Furthermore, the results indicate that future changes in energy demand vary depending on the GCM model, climate change scenario, and regional climatic characteristics. The reduction in heating load showed a consistent trend with the projected warming climate, reflecting a decline in heating demand under increasing mean thermal conditions. Similar decreasing patterns were observed across both GCMs, indicating that heating demand responds relatively directly to warming conditions.

In contrast, cooling demand showed clear differences depending on the climate change scenario. Under the SSP1-2.6 scenario, cooling demand increased in most regions until approximately 2070, after which the rate of increase slowed or stabilized. This suggests that if greenhouse gas mitigation is implemented to some extent, cooling demand may stabilize at a certain level. Under the SSP5-8.5 scenario, however, cooling demand continued to increase throughout the entire projection period, indicating that continued warming without emission reductions could lead to a substantial expansion in cooling demand. Differences between GCMs were also pronounced. In particular, under the UKESM SSP5-8.5 scenario, the annual cooling load in several study regions was projected to exceed 5000 GJ, showing a larger increase compared with projections obtained using the KACE model under the same scenario. The UKESM model is known to be sensitive to aerosol changes and cloud feedback processes, and such characteristics may contribute to relatively higher cooling load projections under warming conditions. This suggests that the magnitude of extreme responses and load increases may vary depending on the structural characteristics of the GCM. Regional comparisons also revealed differences in future energy demand patterns. The Jeju region showed relatively high cooling loads even during the baseline period, and the increase under future scenarios was also larger than in other regions. In addition, several higher-latitude regions showed relatively large increases in cooling demand compared with the baseline period. These results suggest that warming conditions may lead to a rapid expansion of cooling demand in regions where cooling requirements have historically been limited. Overall, the results indicate that while the general direction of energy-load changes under future climate change is consistent, the magnitude and timing of these changes vary depending on the GCM structure, climate change scenario, and regional climate characteristics. These differences highlight the limitations of design decisions based on a single model or scenario and support the need for analytical approaches that incorporate multiple climate projections. At the same time, such structural differences are closely related to the potential reconfiguration of energy demand patterns in livestock housing under future climate conditions.

The projected increase in cooling load has practical implications beyond energy demand alone. In broiler houses, increased cooling requirements are closely related to the need to maintain appropriate indoor thermal conditions during hot periods, which is essential for reducing heat stress, supporting animal welfare, and preventing potential productivity losses or mortality risks. From the farmer’s perspective, a substantial increase in cooling demand may also lead to higher electricity consumption and operational costs, particularly under high-emission climate scenarios. Therefore, the results of this study provide useful information for developing climate-responsive cooling strategies, facility design standards, and energy management plans that support both animal welfare and sustainable broiler production under future climate conditions.

The results of this study indicate a potential shift in the energy demand structure of broiler housing systems from heating-dominated to cooling-dominated under future climate conditions. During the baseline period, heating loads consistently decreased while cooling loads increased substantially. In particular, under the SSP5-8.5 scenario, cooling loads in the late 21st century were projected to increase by more than twofold compared with the baseline period, highlighting the growing importance of cooling load management. These changes suggest that conventional design and heating-cooling operational strategies may need to be re-evaluated under future climate conditions. In addition, the variation in the magnitude of cooling load increases across regions indicates the need for region-specific approaches that account for local climatic characteristics, rather than relying on uniform design standards. These findings emphasize the increasing importance of environmental control in livestock housing under climate change and highlight the need to revise design standards and develop strategies that support sustainable livestock production. The quantitative predictions presented in this study provide a basis for establishing insulation design criteria and cooling management strategies to improve resilience to future climate conditions.

## 5. Conclusions

This study developed an integrated BES-machine learning surrogate framework for predicting heating and cooling energy loads in broiler housing systems and quantitatively analyzed changes in energy demand under future climate change scenarios. The surrogate model was developed using heating and cooling load outputs generated from a previously validated BES model and was subsequently applied to SSP-based future climate data. The model evaluation results showed that XGBoost achieved the best performance for heating load prediction, while Random Forest performed best for cooling load prediction, indicating that the optimal algorithm may vary depending on the characteristics of the target load. The application of climate change scenarios showed a consistent shift toward decreasing heating loads and increasing cooling loads under future climate conditions. In particular, cooling demand was projected to increase substantially under high-emission scenarios, indicating that cooling management will become a critical factor in future energy demand. Although both GCMs showed similar overall trends, differences in the magnitude of energy load changes were observed across models and regions, emphasizing the importance of incorporating regional climatic characteristics into livestock housing design standards.

Overall, this study demonstrates that the proposed machine learning models trained on outputs from physics-based BESs can serve as efficient surrogate models for capturing long-term and region-specific changes in simulated energy demand under climate change. The findings should therefore be interpreted as BES-based energy-load estimates. Nevertheless, the proposed framework provides a scalable analytical framework that reduces the computational burden associated with repeated BES simulations while maintaining predictive accuracy. Furthermore, the results provide a quantitative basis for establishing climate-responsive insulation design criteria and cooling management strategies tailored to regional conditions in broiler housing systems, ultimately serving as a practical foundation for developing livestock housing design standards under future climate conditions. These findings also highlight the need for future cooling management strategies that not only address increasing energy demand but also help maintain appropriate thermal conditions for heat stress prevention, animal welfare, and stable broiler production.

## Figures and Tables

**Table 1:** Annual average temperatures and location information of study areas (2010–2020) (Kwon et al., 2026 [19]).

Study Area	Number of Broilers (Heads, 2020)	Annual Average Temperature (°C) (2010–2020)	Longitude	Latitude
Cheorwon-gun	1,184,678	10.5	127.3042	38.14787
Yangpyeong-gun	2,431,680	12.1	127.4945	37.48863
Chungju-si	978,870	12.0	127.9525	36.97045
Nonsan-si	2,899,189	12.6	127.1082	36.21164
Namwon-si	5,172,657	12.7	127.3965	35.4213
Youngju-si	7,454,525	11.8	128.5169	36.87183
Youngam-gun	2,885,094	13.6	126.6863	34.81666
Sancheong-gun	1,344,562	13.2	127.8791	35.413
Jeju-si	923,774	16.4	126.5297	33.51411

**Table 2:** Categories of variables in the constructed dataset.

Category	Value	Dataset	Number of Variables
Location	Longitude, latitude	Input	2
Year	2011 to 2020		10
Month	January to December		12
Wall thickness	75, 100, 135, 190 mm		4
Roof thickness	130, 180, 220 mm		3
Temperature	tmax_CV, tmin_CV, avg_temp, range_temp		4
Energy load	Heating, cooling	Output	

**Table 3:** Computer Specifications used for modeling.

Module	Specification
CPU	Intel(R)Core i7-9700K CPU @ 3.60 GHz
RAM	64 GB DDR4
Graphics card	Nvidia RTX 2080 Ti
Storage	SSD
Operating system	Windows 10

**Table 4:** Heating model performance evaluation results.

Model (Unit)	XGBoost	Random Forest	Gradient Boosting	Linear Regression
R^2^ (-)	0.999 ± 0.000	0.998 ± 0.000	0.997 ± 0.000	0.915 ± 0.000
RMSE (GJ)	4.34 ± 0.19	6.42 ± 0.23	6.98 ± 0.17	38.98 ± 0.64
MAE (GJ)	3.20 ± 0.10	4.37 ± 0.11	5.29 ± 0.10	29.01 ± 0.41
NMBE (%)	0.01 ± 0.04	−0.01 ± 0.05	0.00 ± 0.07	0.00 ± 0.13
CVRMSE (%)	1.73 ± 0.08	2.55 ± 0.09	2.78 ± 0.06	15.50 ± 0.24
MAPE (%)	1.59 ± 0.05	1.87 ± 0.03	2.62 ± 0.04	14.10 ± 0.25

**Table 5:** Cooling model performance evaluation results.

Model (Unit)	Random Forest	XGBoost	Gradient Boosting	Linear Regression
R^2^ (-)	1.000 ± 0.000	0.999 ± 0.000	0.989 ± 0.001	0.581 ± 0.010
RMSE (GJ)	4.18 ± 0.76	7.63 ± 0.46	24.10 ± 0.65	145.45 ± 3.08
MAE (GJ)	1.41 ± 0.06	4.44 ± 0.15	14.06 ± 0.46	101.67 ± 2.46
NMBE (%)	0.07 ± 0.01	0.02 ± 0.14	0.01 ± 0.57	0.03 ± 1.09
CVRMSE (%)	2.92 ± 0.54	5.32 ± 0.28	16.82 ± 0.24	101.53 ± 2.45
MAPE (%)	8.86 ± 0.62	34.64 ± 2.34	99.77 ± 2.33	926.25 ± 30.35

**Table 6:** Regional classification and minimum insulation standards based on climate characteristics of the study area.

Study Area	Regional Classification Based on Climate Characteristics	Minimum Insulations Standards (mm)	
		Wall	Roof
Cheorwon-gun	Central 1	190	220
Yangpyeong-gun	Central 2	135	220
Chungju-si			
Nonsan-si			
Namwon-si			
Youngju-si			
Youngam-gun	Southern	100	180
Sancheong-gun			
Jeju-si	Jeju	100	180

## Data Availability

The data presented in this study are available from the corresponding author upon reasonable request. The data not publicly available due to their use in an ongoing research project.

## Supplementary Materials

The following supporting information can be downloaded at https://www.mdpi.com/article/10.3390/ani16132097/s1: Table S1: Projected heating energy loads and relative indices under KACE climate change scenarios, applying regional minimum insulation standards; Table S2: Projected cooling energy loads and relative indices under KACE climate change scenarios, applying regional minimum insulation standards; Table S3: Projected heating energy loads and relative indices under UKESM climate change scenarios, applying regional minimum insulation standards; Table S4: Projected cooling energy loads and relative indices under UKESM climate change scenarios, applying regional minimum insulation standards.

## Abbreviations

The following abbreviations are used in this manuscript:

SSP	Shared socioeconomic pathways
BES	Building energy simulation
MAFRA	Ministry of Agriculture, Food and Rural Affairs
IPCC	Intergovernmental Panel on Climate Change
CIMP6	Climate Model Intercomparison Project Phase 6
KMA	Korea Meteorological Administration

## Appendix A

**Table A1:** K-fold validation for heating energy load prediction.

Model	Fold	R^2^	RMSE	MAE	NMBE	CVRMSE	MAPE
XGBoost	1	0.999	4.67	3.37	0.09	1.87	1.67
	2	0.999	4.34	3.22	−0.03	1.73	1.60
	3	0.999	4.40	3.23	−0.03	1.73	1.54
	4	0.999	4.18	3.11	0.00	1.64	1.52
	5	0.999	4.12	3.08	0.02	1.67	1.59
Random Forest	1	0.998	6.60	4.41	0.05	2.64	1.89
	2	0.998	6.62	4.45	−0.02	2.64	1.90
	3	0.998	6.52	4.46	−0.11	2.56	1.90
	4	0.998	6.37	4.39	0.01	2.49	1.84
	5	0.998	5.99	4.15	0.03	2.42	1.84
Gradient Boosting	1	0.997	6.95	5.21	0.10	2.78	2.62
	2	0.997	7.26	5.40	0.05	2.90	2.66
	3	0.997	7.00	5.23	−0.12	2.75	2.56
	4	0.997	7.00	5.42	−0.01	2.74	2.59
	5	0.997	6.71	5.21	0.00	2.71	2.67
Linear Regression	1	0.919	37.96	28.50	−0.21	15.22	14.12
	2	0.916	39.59	29.02	0.12	15.79	14.01
	3	0.914	38.76	29.00	−0.04	15.22	13.68
	4	0.912	39.75	29.75	0.15	15.57	14.24
	5	0.914	38.82	28.80	−0.01	15.69	14.43

**Table A2:** K-fold validation for cooling energy load prediction.

Model	Fold	R^2^	RMSE	MAE	NMBE	CVRMSE	MAPE
XGBoost	1	0.999	8.52	4.57	0.13	5.78	37.40
	2	0.999	7.44	4.61	−0.23	5.11	31.38
	3	0.999	7.57	4.36	−0.06	5.50	37.06
	4	0.999	7.24	4.20	0.10	5.19	34.56
	5	0.999	7.38	4.47	0.16	5.04	32.81
Random Forest	1	1.000	5.23	1.45	−0.11	3.55	9.02
	2	1.000	2.94	1.31	0.04	2.02	8.72
	3	1.000	4.62	1.39	0.15	3.36	8.47
	4	1.000	4.17	1.41	0.18	2.99	9.95
	5	1.000	3.92	1.51	0.08	2.68	8.14
Gradient Boosting	1	0.988	25.25	14.82	0.65	17.12	103.94
	2	0.989	24.04	14.09	−0.68	16.52	96.76
	3	0.989	23.44	13.66	−0.59	17.03	99.29
	4	0.989	23.53	13.52	0.02	16.87	99.75
	5	0.988	24.25	14.22	0.63	16.56	99.10
Linear Regression	1	0.569	149.52	105.14	0.90	101.38	942.51
	2	0.576	146.16	102.97	−1.15	100.41	932.05
	3	0.599	140.61	97.66	1.37	102.17	929.70
	4	0.579	147.31	101.07	0.35	105.60	958.04
	5	0.585	143.62	101.51	−1.34	98.11	868.93
