# Supplementary material for: BES-Driven Machine Learning Prediction of Future Energy Loads in Broiler Housing Under SSP Climate Scenarios in South Korea
Source: Animals (Basel). 2026 Jul 6;16(13):2097. doi: 10.3390/ani16132097 (PMC13359958; doi:10.3390/ani16132097)
Supplement: Supplementary file 1 [file animals-16-02097-s001.zip › animals-4383182-supplementary.pdf]

Table S1. Projected heating energy loads and relative indices under KACE climate change scenarios, applying regional minimum insulation standards

			2011-2020	2011-2040	2041-2070	2071-2100
Central 1	Cheorwon	SSP1-2.6	2,382.5	2,226.7	2,146.3	2,139.1
			100.0	93.5	90.1	89.8
		SSP5-8.5	2,382.5	2,232.1	2,098.6	1,947.8
			100.0	93.7	88.1	81.8
Central 2	Yangpyeong	SSP1-2.6	2,578.8	2,497.1	2,428.5	2,427.0
			100.0	96.8	94.2	94.1
		SSP5-8.5	2,578.8	2,498.6	2,375.3	2,234.0
			100.0	96.9	92.1	86.6
	Chungju	SSP1-2.6	2,587.4	2,471.1	2,389.8	2,384.1
			100.0	95.5	92.4	92.1
		SSP5-8.5	2,587.4	2,474.6	2,339.5	2,188.3
			100.0	95.6	90.4	84.6
	Nonsan	SSP1-2.6	2,588.5	2,416.4	2,341.8	2,340.9
			100.0	93.4	90.5	90.4
		SSP5-8.5	2,588.5	2,423.8	2,285.6	2,141.5
			100.0	93.6	88.3	82.7
	Namwon	SSP1-2.6	2,528.1	2,406.6	2,317.6	2,315.7
			100.0	95.2	91.7	91.6
		SSP5-8.5	2,528.1	2,410.1	2,258.1	2,106.1
			100.0	95.3	89.3	83.3
	Youngju	SSP1-2.6	2,615.6	2,494.7	2,416.8	2,408.0
			100.0	95.4	92.4	92.1
		SSP5-8.5	2,615.6	2,496.5	2,360.8	2,208.0
			100.0	95.4	90.3	84.4
Southern	Youngam	SSP1-2.6	2,937.2	2,649.0	2,533.5	2,518.2
			100.0	90.2	86.3	85.7
		SSP5-8.5	2,937.2	2,654.1	2,446.2	2,189.5
			100.0	90.4	83.3	74.5
	Sangcheong	SSP1-2.6	3,029.2	2,826.1	2,710.8	2,699.0
			100.0	93.3	89.5	89.1
		SSP5-8.5	3,029.2	2,832.5	2,631.3	2,435.6
			100.0	93.5	86.9	80.4
Jeju	Jeju	SSP1-2.6	3,269.5	2,930.2	2,676.0	2,648.5
			100.0	89.6	81.8	81.0
		SSP5-8.5	3,269.5	2,941.0	2,515.4	2,084.0
			100.0	90.0	76.9	63.7

Note: Energy loads are presents as absolute values (GJ) and as relative indices normalized to the baseline period (2011-2020 = 100). Regional minimum insulation standards were applied according to the applicable building energy codes for each location.

Table S2. Projected cooling energy loads and relative indices under KACE climate change scenarios, applying regional minimum insulation standards

			2011-2020	2011-2040	2041-2070	2071-2100
Central 1	Cheorwon	SSP1-2.6	1,412.2	2858.5	3579.0	3552.9
			100.0	202.4	253.4	251.6
		SSP5-8.5	1,412.2	2751.9	4014.3	4903.6
			100.0	194.9	284.3	347.2
Central 2	Yangpyeong	SSP1-2.6	1,823.7	2642.0	3027.3	2905.8
			100.0	144.9	166.0	159.3
		SSP5-8.5	1,823.7	2553.0	3613.0	4506.1
			100.0	140.0	198.1	247.1
	Chungju	SSP1-2.6	1,865.5	2587.8	3145.4	3108.5
			100.0	138.7	168.6	166.6
		SSP5-8.5	1,865.5	2560.3	3689.1	4625.4
			100.0	137.2	197.8	247.9
	Nonsan	SSP1-2.6	1,754.0	2902.4	3377.2	3410.5
			100.0	165.5	192.5	194.4
		SSP5-8.5	1,754.0	2843.6	3835.7	4590.4
			100.0	162.1	218.7	261.7
	Namwon	SSP1-2.6	1,827.3	2966.2	3556.9	3563.9
			100.0	162.3	194.7	195.0
		SSP5-8.5	1,827.3	2985.4	4071.5	4954.6
			100.0	163.4	222.8	271.1
	Youngju	SSP1-2.6	1,451.2	2330.4	2814.8	2765.5
			100.0	160.6	194.0	190.6
		SSP5-8.5	1,451.2	2320.2	3363.0	4405.7
			100.0	159.9	231.7	303.6
Southern	Youngam	SSP1-2.6	1,632.6	3125.2	3747.8	3783.4
			100.0	191.4	229.6	231.7
		SSP5-8.5	1,632.6	3097.6	4110.3	5146.0
			100.0	189.7	251.8	315.2
	Sangcheong	SSP1-2.6	1,686.6	2865.0	3522.3	3471.8
			100.0	169.9	208.8	205.8
		SSP5-8.5	1,686.6	2872.8	4017.3	4776.2
			100.0	170.3	238.2	283.2
Jeju	Jeju	SSP1-2.6	2,144.1	4004.3	5163.9	5232.1
			100.0	186.8	240.8	244.0
		SSP5-8.5	2,144.1	3987.9	5699.1	7006.3
			100.0	186.0	265.8	326.8

Note: Energy loads are presents as absolute values (GJ) and as relative indices normalized to the baseline period (2011-2020 = 100). Regional minimum insulation standards were applied according to the applicable building energy codes for each location.

Table S3. Projected heating energy loads and relative indices under UKESM climate change scenarios, applying regional minimum insulation standards

			2011-2020	2011-2040	2041-2070	2071-2100
Central 1	Cheorwon	SSP1-2.6	2,382.5	2,279.7	2,221.8	2,196.8
			100.0	95.7	93.3	92.2
		SSP5-8.5	2,382.5	2,295.0	2,137.8	1,977.4
			100.0	96.3	89.7	83.0
Central 2	Yangpyeong	SSP1-2.6	2,578.8	2,505.9	2,421.4	2,379.8
			100.0	97.2	93.9	92.3
		SSP5-8.5	2,578.8	2,525.6	2,312.0	2,085.6
			100.0	97.9	89.7	80.9
	Chungju	SSP1-2.6	2,587.4	2,503.2	2,434.6	2,403.7
			100.0	96.7	94.1	92.9
		SSP5-8.5	2,587.4	2,521.1	2,326.6	2,128.4
			100.0	97.4	89.9	82.3
	Nonsan	SSP1-2.6	2,588.5	2,428.7	2,336.1	2,296.4
			100.0	93.8	90.3	88.7
		SSP5-8.5	2,588.5	2,451.0	2,215.8	1,960.8
			100.0	94.7	85.6	75.8
	Namwon	SSP1-2.6	2,528.1	2,400.9	2,304.9	2,258.2
			100.0	95.0	91.2	89.3
		SSP5-8.5	2,528.1	2,423.9	2,169.3	1,883.0
			100.0	95.9	85.8	74.5
	Youngju	SSP1-2.6	2,615.6	2,526.9	2,454.1	2,422.6
			100.0	96.6	93.8	92.6
		SSP5-8.5	2,615.6	2,543.2	2,344.2	2,148.6
			100.0	97.2	89.6	82.1
Southern	Youngam	SSP1-2.6	2,937.2	2,664.8	2,537.0	2,469.8
			100.0	90.7	86.4	84.1
		SSP5-8.5	2,937.2	2,698.1	2,339.1	1,899.3
			100.0	91.9	79.6	64.7
	Sangcheong	SSP1-2.6	3,029.2	2,869.9	2,773.2	2,725.4
			100.0	94.7	91.6	90.0
		SSP5-8.5	3,029.2	2,891.8	2,620.1	2,343.8
			100.0	95.5	86.5	77.4
Jeju	Jeju	SSP1-2.6	3,269.5	3,094.5	2,935.1	2,868.9
			100.0	94.6	89.8	87.7
		SSP5-8.5	3,269.5	3,156.6	2,663.3	2,170.9
			100.0	96.5	81.5	66.4

Note: Energy loads are presents as absolute values (GJ) and as relative indices normalized to the baseline period (2011-2020 = 100). Regional minimum insulation standards were applied according to the applicable building energy codes for each location.

Table S4. Projected cooling energy loads and relative indices under UKESM climate change scenarios, applying regional minimum insulation standards

			2011-2020	2011-2040	2041-2070	2071-2100
Central 1	Cheorwon	SSP1-2.6	1,412.2	2494.8	3281.5	3451.6
			100.0	176.7	232.4	244.4
		SSP5-8.5	1,412.2	2538.8	4056.1	5390.9
			100.0	179.8	287.2	381.7
Central 2	Yangpyeong	SSP1-2.6	1,823.7	2805.9	3574.1	3775.7
			100.0	153.9	196.0	207.0
		SSP5-8.5	1,823.7	2888.1	4342.1	5590.4
			100.0	158.4	238.1	306.5
	Chungju	SSP1-2.6	1,865.5	2467.6	3161.5	3409.6
			100.0	132.3	169.5	182.8
		SSP5-8.5	1,865.5	2571.1	3932.6	5300.8
			100.0	137.8	210.8	284.2
	Nonsan	SSP1-2.6	1,754.0	3068.8	3776.1	3956.6
			100.0	175.0	215.3	225.6
		SSP5-8.5	1,754.0	3084.2	4430.9	5815.6
			100.0	175.8	252.6	331.6
	Namwon	SSP1-2.6	1,827.3	3145.2	3901.4	4055.9
			100.0	172.1	213.5	222.0
		SSP5-8.5	1,827.3	3184.2	4612.2	6164.9
			100.0	174.3	252.4	337.4
	Youngju	SSP1-2.6	1,451.2	2146.6	2858.7	3043.4
			100.0	147.9	197.0	209.7
		SSP5-8.5	1,451.2	2297.4	3679.8	5208.1
			100.0	158.3	253.6	358.9
Southern	Youngam	SSP1-2.6	1,632.6	2980.5	3651.8	3840.2
			100.0	182.6	223.7	235.2
		SSP5-8.5	1,632.6	3028.4	4365.9	5834.5
			100.0	185.5	267.4	357.4
	Sangcheong	SSP1-2.6	1,686.6	2875.9	3509.7	3715.0
			100.0	170.5	208.1	220.3
		SSP5-8.5	1,686.6	2904.3	4217.6	5804.6
			100.0	172.2	250.1	344.2
Jeju	Jeju	SSP1-2.6	2,144.1	3325.0	4008.3	4229.0
			100.0	155.1	186.9	197.2
		SSP5-8.5	2,144.1	3294.2	5078.6	6660.6
			100.0	153.6	236.9	310.6

Note: Energy loads are presents as absolute values (GJ) and as relative indices normalized to the baseline period (2011-2020 = 100). Regional minimum insulation standards were applied according to the applicable building energy codes for each location.
